# Case Report: Surgical androgen deprivation therapy for prostate cancer in a patient with concurrent scrotal paget’s disease

**DOI:** 10.3389/fonc.2025.1509285

**Published:** 2025-06-26

**Authors:** Sinan Yang, Qiao Wang, Yuanlong Shi, Bo Peng, Jinye Yang, Zongyan Luo, Can Li, Jian Xu, Wei Luo, Chengwei Bi, Bin Zhao, Yong Yang

**Affiliations:** The Third Affiliated Hospital of Kunming Medical University, Yunnan Cancer Hospital, Peking University Cancer Hospital Yunnan, Department of Urology II, Kunming, China

**Keywords:** prostate cancer, scrotal paget’s disease, extramammary paget’s disease, bilateral orchiectomy, androgen deprivation therapy

## Abstract

**Introduction:**

Prostate cancer (PCa) and Scrotal Paget’s disease (SPD) are two distinct malignancies, and reports of their concurrent occurrence are very limited. The aim of this case was to discuss the individualized treatment strategy for an elderly patient with metastatic PCa combined with SPD.

**Case presentation:**

An octogenarian male (aged 88 years) with metastatic PCa (Gleason 8 = 4 + 4, bone metastases, suspected lung involvement) received androgen deprivation therapy (ADT: bicalutamide + goserelin), achieving biochemical control (PSA <0.1 ng/mL; testosterone <20 ng/dL) over three years (2020-2023). In September 2023, he developed a painless scrotal nodule (0.5 cm), which progressed to a 3 cm ulcerated lesion with pruritus and bleeding by December 2023. Histopathology confirmed SPD. After multidisciplinary review and family prioritization of symptom relief, wide local excision (3-cm margins) and bilateral orchiectomy were performed on December 9, 2023. Postoperative pathology confirmed a primary SPD (CK7+/GCDFP-15+). Postoperative recovery was uncomplicated, and no recurrence was observed at the one-year follow-up in December 2024.

**Clinical discussion:**

Dual pathology requires a multimodal approach. Surgery can control symptoms, simplify ADT, and reduce the risk of SPD recurrence. This decision requires balancing tumor efficacy, age, and quality of life (QoL).

**Conclusion:**

Combined surgical resection and ADT may benefit older patients with synchronous PCa and SPD(CK7+/GCDFP-15+), but patient selection and informed consent remain critical.

PCa is the most common malignant tumor of the male genitourinary system. According to the World Health Organization (WHO) GLOBOCAN 2020 report, PCa is the second most frequently diagnosed malignancy in men worldwide, following lung cancer. Abnormalities are typically detected through diagnostic rectal examination (DRE) or PSA screening; however, a definitive diagnosis requires histopathologic analysis via prostate biopsy. The primary treatment for PCa is radical prostatectomy (RP), though other options such as radiation therapy and ADT may also be utilized (1). Extramammary Paget’s disease (EMPD) is a rare malignancy that typically arises in areas with apocrine glands, such as the vulva, scrotum, and penis, with scrotal EMPD being less common compared to vulvar and perianal types (2). Clinically, EMPD often presents as infiltrative erythema, which can mimic other skin conditions such as eczema. Scrotal EMPD has a high rate of metastasis and recurrence, leading to extensive spread of tumor cells once they invade the dermis.In this paper, we present a case of PCa co-occurring with SPD. The patient was admitted to the Department of Urology at Yunnan Cancer Hospital, where an individualized treatment approach was implemented.

## Case report

An 88-year-old man with high socioeconomic status and a stable family environment presented with progressive urinary symptoms (nocturia, hematuria), bone pain and dyspnea. He denies smoking, alcohol or drug use. He reported anxiety and depressive symptoms, but had no formal psychiatric diagnosis. There was no genetic or oncologic disease in the family history. Comorbidities included hypertension (20+ years), coronary artery disease (10+ years), and cholelithiasis (3 years).

**Figure 1:**
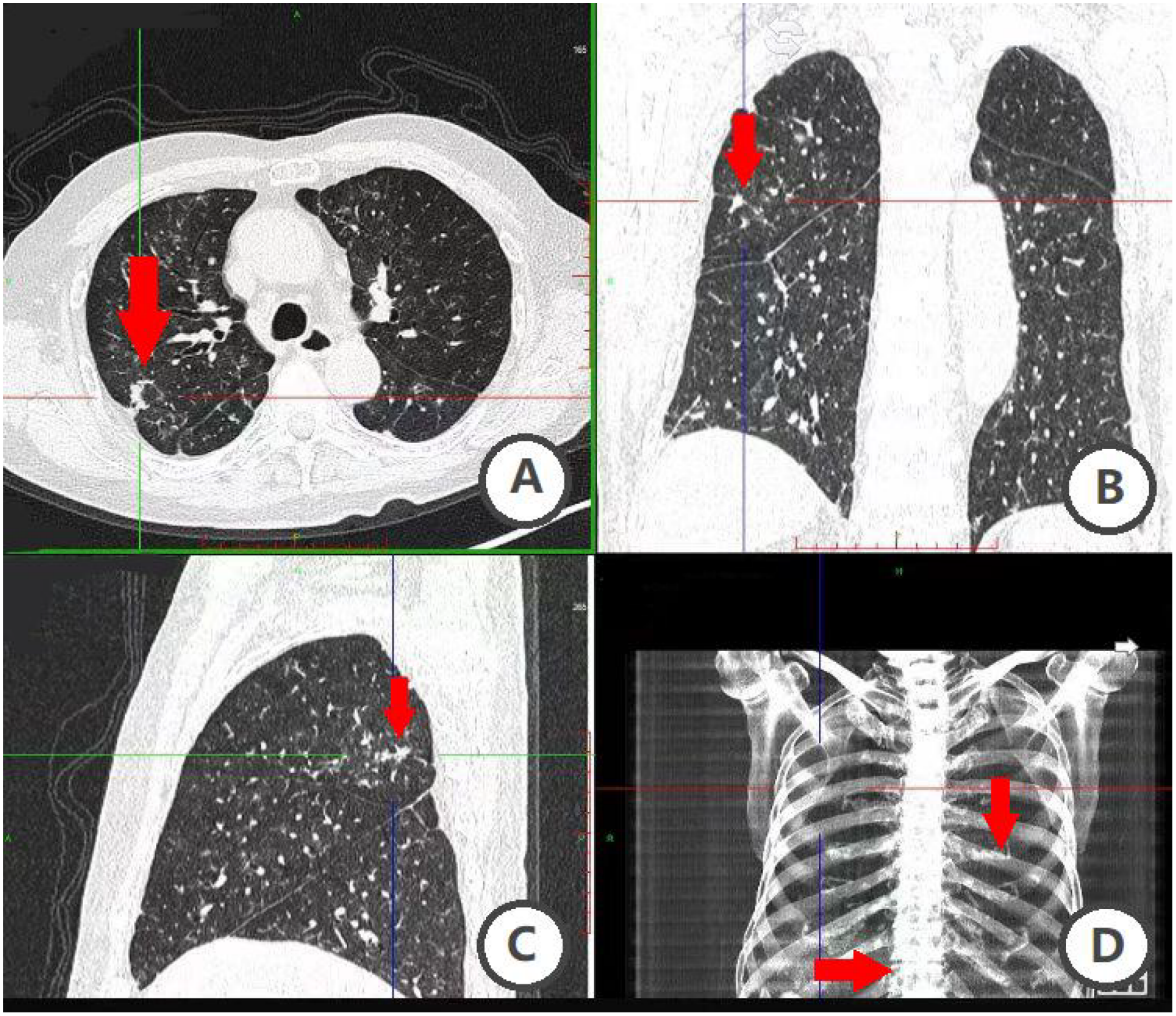
Unenhanced CT combined with 3D post-processing. **(A–D)** figure suggests that the patient has multiple lung nodules and metastasis is possible; uneven bone density of ribs and thoracic vertebrae.

**Figure 2:**
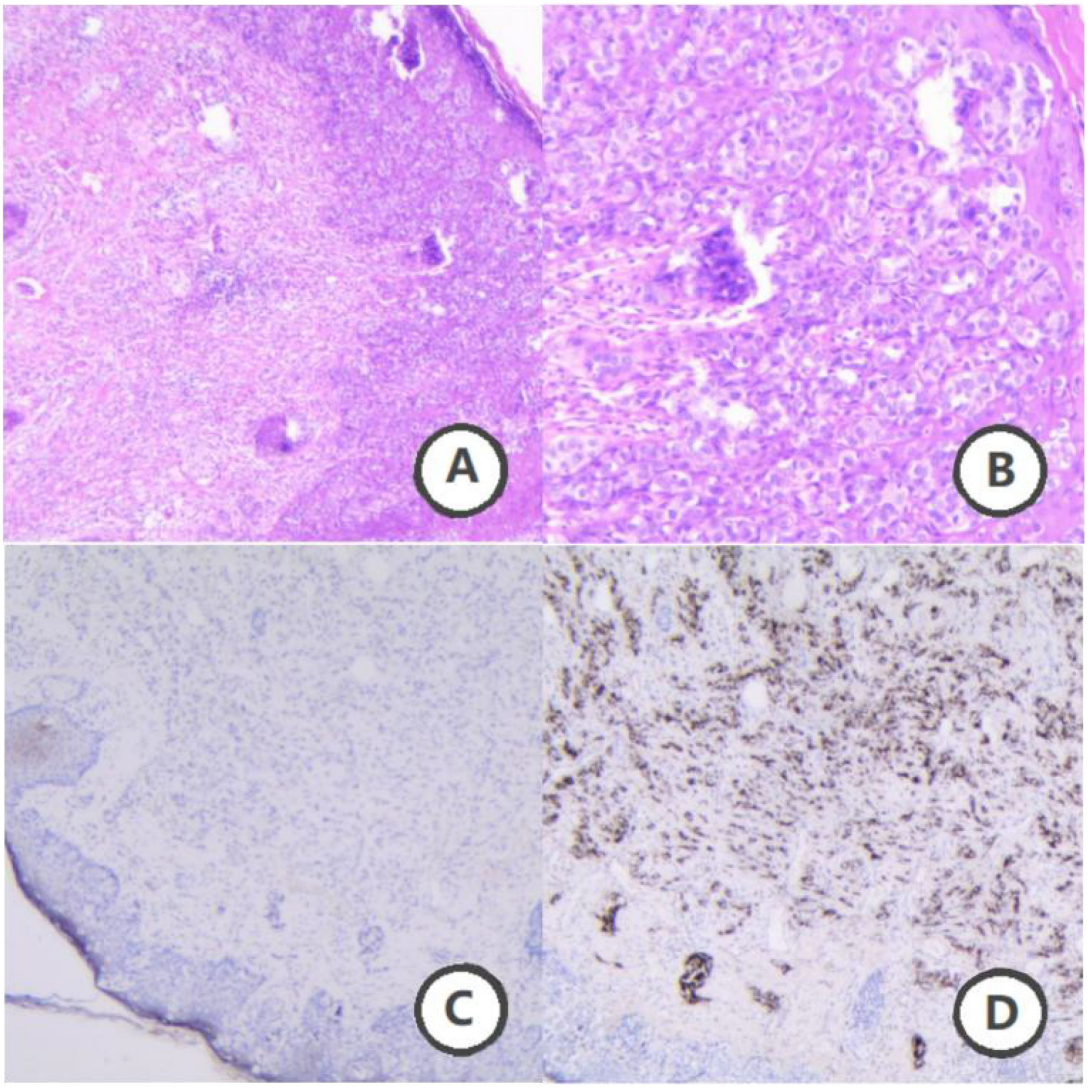
Pathological findings of scrotal skin lesions. **(A)** Hematoxylin and eosin staining (H&E, ×40 magnification). **(B)** (H&E, ×100 magnification), Paget cells were observed, characterized by their large, heterogeneous size with prominent nuclei and nucleoli. These cells exhibit abundant cytoplasm with eosinophilic light staining. The tumor cells are dispersed throughout the epidermis in single cells or small clusters. **(C)** Immunohistochemical staining (IHC, ×40 magnification). **(D)** (IHC, ×40 magnification) Immunohistochemistry reveals positivity for CK, CK7, and CEA, with CK20 showing focal weak positivity. Ki67 is positive at approximately 30%. Additionally, GCDFP-15 is positive. These findings suggest primary Paget’s disease of the scrotum, with prostate origin being excluded.

In January 2020, an examination at an outside hospital suggested elevated PSA levels. A prostate biopsy was performed and the pathology results were suggestive of prostatic adenocarcinoma (Gleason score 8 = 4 + 4). Bone scan suggested multiple abnormal metabolic activity in the thoracic spine and ribs, suggesting possible bone metastasis from prostate cancer. Follow-up unenhanced CT combined with 3D post-processing (**Figure 1**) in April 2020 at our hospital showed a prostate malignant tumor with metastasis to the thoracic spine and ribs, and possible lung metastasis. Because of the patient’s advanced age and imaging suggesting bone and possible lung metastasis, surgical treatment was not recommended, and combined endocrine therapy with bicalutamide and goserelin was administered for over three years, with regular monitoring of PSA and testosterone levels, and with regular reviews confirming effective disease control. In September 2023, the patient found a hard cauliflower-like mass in the scrotum near the root of the penis with a diameter of about 0.5 cm, accompanied by erythema and swelling, but no pain. The patient initially neglected the lesion, but it progressively enlarged, expanding on the scrotum to approximately 3 x 2 cm. Symptoms progressively worsened, including pruritus, ulceration, white discharge, and slight oozing of blood. The patient said that the symptoms (itching, ulceration and bleeding) were upsetting him and that his family prioritized improving his QoL and requested surgical intervention. Consequently, he underwent wide local excision of the scrotal lesion and bilateral orchiectomy on December 9, 2023. Postoperative pathologic examination of the scrotal skin lesion (**Figure 2**) showed a poorly differentiated adenocarcinoma, which was of cutaneous origin and not of prostatic origin. The patient’s prognosis was good, and symptomatic treatment was continued until now. The patient reported no significant discomfort, and his QoL had improved compared to his preoperative baseline, and he was followed up regularly, with no obvious progression of the disease. The patient's main diagnosis and treatment timeline is as follows (**Table 1**).

**Table 1:** Timeline of the patient's main diagnosis and treatment.

Date	Event
2020-01	Elevated PSA; prostate biopsy confirms Gleason 8 = 4 + 4 PCa. Bone scan: metastases.
2020-04	PET-CT suggests possible lung metastases.
2020-2023	ADT (bicalutamide + goserelin): PSA <0.1 ng/mL.
2023-06	0.5 cm scrotal nodule detected.
2023-09	Lesion progresses to 3 cm with ulceration/bleeding. SPD has been confirmed.
2023-12-09	Wide excision (3-cm margins) + bilateral orchiectomy.
2024-12	No recurrence at 1-year follow-up.

PET TC imaging performed at the time of the suspected cutaneous relapse has been lost. Moreover, according to patient preference, no further imaging was carried out and thus available.

## Discussion

Prostate cancer (PCa) is one of the most common malignancies affecting men globally, ranking second in incidence and fifth in mortality among male cancers. Treatment options include surgery, radiotherapy, endocrine therapy, and chemotherapy. Among androgen deprivation therapies (ADT), surgical castration (bilateral orchiectomy) is a palliative approach for PCa. In developing countries, bilateral orchiectomy may be a preferred treatment option due to considerations of cost, accessibility, patient compliance, and frequency of required healthcare visits (3). Historically, bilateral orchiectomy has been a crucial method for managing locally advanced PCa. This procedure is straightforward, low-cost, and associated with minimal side effects, and can be performed under local or general anesthesia. Post-surgery, serum testosterone levels decrease rapidly, typically reaching castrate levels within 12 hours. Bilateral orchiectomy is especially suitable when rapid testosterone reduction is needed or when pharmacologic options are financially or logistically challenging. However, compared to pharmacological approaches, surgical orchiectomy can have negative physical and psychological effects on patients. With advancements in medical technology, patients who are reluctant to undergo testicular resection now have the option of pharmacological alternatives. These treatments inhibit gonadotropin release from the pituitary gland, thereby reducing androgen secretion from the testes. Common medications used include luteinizing hormone-releasing hormone (LHRH) agonists or antagonists. This “medical castration” has become the primary treatment for progressive PCa. As a result, bilateral orchiectomy, once a standard treatment, has been largely supplanted by less invasive hormonal therapies. Its indications are now primarily limited to the removal of atrophied testes or those damaged by trauma, torsion, infection, or tumor (4). Clinical studies have shown that medical castration is as effective as surgical castration in lowering testosterone levels and prolonging patient survival, with no significant differences in outcomes.

In this case, after the diagnosis of PCa, due to the consideration of the patient’s advanced age and physical condition, he underwent pharmacological ADT for more than 3 years, and his PSA level was well controlled. However, the patient was subsequently diagnosed with extensive SPD, and the patient expressed a “desire to resolve the scrotal symptoms as soon as possible.” The patient’s family strongly demanded that “improvement of QoL should be prioritized.” After a multidisciplinary consultation and obtaining the patient’s full informed consent, our department decided to implement a personalized treatment plan, including scrotal lesion excision and bilateral orchiectomy. This surgery not only removed the scrotal Paget lesion, but also provided surgical castration for the PCa and mitigated risks of SPD recurrence and metastasis of SPD.

SPD, also known as EMPD of the scrotum, was first described by Crocker in 1889. This rare malignant tumor of the genitourinary system primarily affects men aged 60 to 70 years. It is classified as a type of EMPD, with an uncertain etiology possibly related to inflammation, chronic irritation, and human papillomavirus type 16 infection. Pathological examination reveals large, lightly stained abnormal cells (Paget cells) within the epidermis, a distinct type of carcinoma. Its clinical manifestations, which resemble those of psoriasis, eczematous dermatitis, and fungal infections, often lead to underdiagnosis and misdiagnosis, resulting in treatment delays. The disease typically has a prolonged history, presenting as itchy skin, well-defined erythema with or without hyperpigmentation, and erythematous lesions that may crust, scale, or progress to nodules, vesicles, or deep ulcers in advanced stages. Approximately 10% of patients may be asymptomatic (5).

The high metastasis and recurrence rates of scrotal EMPD can lead to extensive tumor spread once it invades the dermis. Typically, the postoperative recurrence rate ranges from 15% to 33%. About 10% of recurrent cases may progress to invasive carcinoma or metastasize, complicating treatment further. Follow-up studies of patients who underwent successful surgery for primary EMPD indicate a postoperative recurrence rate of 18.4%, with 34 patients experiencing recurrence. Among these, 18 patients died from the disease, resulting in a 5-year survival rate of 52.5% for those with recurrence (6).

Currently, the most commonly used biomarkers for the pathological confirmation of EMPD are CK7 and GATA3. In our patient, immunohistochemical analysis showed CK7 positivity, which is consistent with existing reports. Recent studies have identified TRPS1 as a sensitive and specific biomarker for EMPD. TRPS1 may also be useful in excluding secondary vulvar involvement in cases of uroepithelial and anorectal cancers (7).

In this case, the scrotal localization of the lesion mandated extensive surgical resection of both the scrotal lesion and bilateral testes as part of a multimodal strategy for concurrent PCa management. While wide surgical excision (requiring 2–3 cm margins beyond visible lesions and full-thickness scrotal resection) remains the standard for SPD, clinical decisions must balance risks and benefits tailored to individual circumstances. The adopted protocol achieved symptomatic relief through complete lesion excision and bilateral orchiectomy, while simultaneously alleviating the long-term pharmacologic burden of PCa management via surgical castration—a particularly pragmatic approach for elderly patients with metastatic disease. However, the intervention’s inherent risks (anesthetic complications, extensive tissue loss impacting wound repair) underscore its limitations in geriatric populations.

Compared to surgical intervention, conservative observation or local pharmacotherapy—while avoiding procedural trauma—often fails to control progressive lesions. In this case, the rapid progression of a scrotal lesion from 0.5 cm to 3 cm with ulceration and hemorrhage within three months of initial non-surgical management (as advised by an external institution) underscores the limitations of such approaches for active disease. Radiotherapy, though occasionally used adjunctively, is constrained by the poor radiation tolerance of scrotal skin, which risks exacerbating ulceration. Emerging therapies, such as anti-HER2 agents and PD-1 inhibitors, have demonstrated preliminary promise in case reports (12); however, their efficacy remains supported only by small-scale studies, precluding their recommendation as first-line therapies at present.

Recurrence remains a significant concern in EMPD despite surgical intervention. For recurrent lesions, topical imiquimod therapy has demonstrated efficacy, with studies reporting favorable response rates and tolerability (8, 9). Notably, 5% imiquimod cream may serve as a non-invasive alternative to repeat excision (10). In two cases of recurrent, generalized EMPD, complete remission was achieved using 20% photodynamic therapy (PDT) with 5-aminolevulinic acid (ALA) and imiquimod (11). Immune checkpoint inhibitors targeting the programmed cell death receptor (PD-1) and/or its ligand (PD-L1), approved for various malignancies, also show emerging potential in EMPD management, as evidenced by preclinical and clinical studies (12).

In 1994, a case of concurrent SPD and PCa was documented in which the patient underwent carboplatin-paclitaxel chemotherapy. This regimen achieved initial partial remission but culminated in disease progression. While the therapeutic strategy was guided by the adenocarcinoma’s biological profile, clinical efficacy remained constrained. The patient demonstrated an exceptionally poor prognosis, exhibiting a median survival under 12 months before succumbing to widespread systemic metastases (including osseous, pulmonary, and cerebral involvement) (13).

Cases of synchronized PCa and scrotal medullary lesions SPD are extremely rare. In this case, an elderly patient with high-volume metastatic PCa underwent individualized surgical treatment consisting of a localized wide excision of the scrotal lesion (margins 3 cm) and bilateral orchiectomy. This decision was based on the patient’s symptom burden (itching, ulceration, bleeding) and the family’s priority to improve QoL. Three days after the operation, the patient’s vital signs remained stable, and the patient complained that his scrotal symptoms had disappeared without any particular discomfort. At the one-year follow-up (December 2024), no complications or disease progression were noted. However, clinicians must weigh the irreversibility of this intervention (including its physical and psychological consequences) against its benefits. Adapting treatment strategies to the individual clinical situation remains essential to optimize survival and QoL.

## Data Availability

The raw data supporting the conclusions of this article will be made available by the authors, without undue reservation.
